# Chinese herbal formula Xuefu Zhuyu for primary dysmenorrhea patients (CheruPDYS): a study protocol for a randomized placebo-controlled trial

**DOI:** 10.1186/s13063-021-05050-w

**Published:** 2021-01-26

**Authors:** Geng Li, Zhe Zhang, Li Zhou, Shaojun Liao, Jing Sun, Yinghua Liu, Xin Wang, Zehuai Wen

**Affiliations:** 1 The Second Affiliated Hospital of Guangzhou University of Chinese Medicine, Guangzhou, Guangdong China; 2 Key Unit of Methodology in Clinical Research, Guangdong Provincial Hospital of Chinese Medicine, Guangzhou, Guangdong China; 3 Liaoning University of Traditional Chinese Medicine, Shenyang, Liaoning China; 4 The Second Clinical College of Guangzhou University of Chinese Medicine, Guangzhou, Guangdong China; 5 Department of Obstetrics and Gynecology, Affiliated Hospital of Liaoning University of Traditional Chinese Medicine, Shenyang, Liaoning China; 6 Guangdong Provincial Key Laboratory of Clinical Research on Traditional Chinese Medicine Syndrome, Guangzhou, Guangdong China; 7 State Key Laboratory of Dampness Syndrome of Chinese Medicine, The Second Affiliated Hospital of Guangzhou University of Chinese Medicine, Guangzhou, Guangdong China

**Keywords:** Chinese herbal medicine, Primary dysmenorrhea, Xuefu Zhuyu Oral Liquid, Placebo, Randomized controlled trial

## Abstract

**Background:**

Epidemiological studies have shown that young women often suffer from primary dysmenorrhea (PD) which is a common cause that affects their routine work and quality of life. Chinese herbal medicine has been widely used for PD in China. A systematic review found that Xuefu Zhuyu (XFZY) has a promising effect on PD management, yet there is a dearth of high-quality evidence in support of this claim. We want to conduct a randomized controlled trial to evaluate the efficacy and safety of XFZY for PD patients.

**Methods:**

This is a protocol for a multicenter, randomized, placebo-controlled trial. A total of 248 participants with PD will be recruited at 6 centers and randomized into two groups—a herbal treatment group and a placebo group. The participants will receive either XFZY or placebo, three times per day, for 3 menstrual cycles, with a 12-week follow-up. The primary outcome will be the mean change in pain intensity as measured by VAS, while the change in menstrual pain duration, the change in peak pain intensity as measured by VAS, the Cox Menstrual Symptom Scale (CMSS), quality of life EQ-5D-5L, cumulative painkiller consumption, and health economics will be included as secondary outcomes. Adverse events will also be reported.

**Discussion:**

This protocol describes a multicenter, double-blind, randomized, placebo-controlled trial that investigates the efficacy and safety of XFZY for primary dysmenorrhea. Validated evaluation tools will assess dysmenorrhea severity. We believe that this research will provide important evidence regarding the use of XFZY to treat dysmenorrhea.

**Trial registration:**

Chinese Clinical Trial Registry ChiCTR1900026819. Registered on 23 October 2019

## Background

Dysmenorrhea is a common gynecological disease. Its prevalence ranges from 50 to 90% across ethnicities and geographical locations [1]. Dysmenorrhea can be classified into primary dysmenorrhea (PD) and secondary dysmenorrhea, with the former more prevalent in young women [2]. More than one half of PD patients are moderate or severe among Chinese Female University students [3], diminishing young females’ quality of life [3, 4], restricting their daily activities, and causing absenteeism from work and school [3, 4]. Despite its high incidence, most patients do not seek medical help for dysmenorrhea [5].

Non-steroidal anti-inflammatory drugs (NSAIDs) are an effective treatment for PD [5, 6], but can cause adverse events in the gastrointestinal tract and central nervous system [7]. These include indigestion, headaches, and drowsiness [6]. Hormone therapy generally requires periodic, prolonged, or continuous use [5], and patients may find it inconvenient. Therefore, some patients also seek complementary and alternative therapies, including Chinese medicine (CM), to treat PD.

Physicians of Chinese medicine treat patients according to pattern differentiation. Pattern (*Zheng* or syndrome in Chinese medicine) is a diagnostic conclusion based on pathological changes in a disease at a certain stage. It includes features such as the nature of the disease, cause, location, and development trends [8]. *Qi stagnation* and *blood stasis pattern* (*QBP*) are common PD patterns [9–11]. Its features always present as distending pain and stabbing pain [12]. The CHM formula Xuefu Zhuyu (XFZY) has been widely used in clinical in China [13] and was the most frequently used in research focused on blood stasis in Korea [14]. It comes as both an oral liquid and a capsule [15]. XFZY promotes *Qi* and activates the *blood* to relieve *QBP* symptoms [12].

Pharmacological experiments have found that XFZY restrains platelet aggregation induced by adenosine diphosphate, arachidonic acid, and collagen [16]. This has been shown to inhibit thrombogenesis in rats [16, 17] and increase the number of open capillaries [18]. Thus, it can be inferred that XFZY can treat the pathogenesis of QBP.

XFZY was approved by the China Food and Drug Administration (CFDA) in 2002 [19] and has been included in the Chinese pharmacopeia [20]. XFZY’s components include Persicae semen (Taoren), Carthami flos (Honghua), Rehmanniae radix (Dihuang), Angelicae sinensis radix (Danggui), Chuanxiong rhizome (Chuanxiong), Paeoniaeradix rubra (Chishao), Achyranthis bidentatae radix (Niuxi), Platycodonis radix (Jiegeng), Bupleuri radix (Chaihu), Aurantii fructus (Zhiqiao) fried with bran, and Glycyrrhizae radix et rhizome (Gancao).

A recent systematic review [21] revealed that XFZY has a promising effect on PD management. Some trials also found the efficacy of XFZF on PD. For example, a randomized clinical trial (RCT) [22] found that XFZY had a higher cured rate and a lower recurrence rate compared with indomethacin for PD. Some other trials also found XFZY had a better response rate for PD as compared to conventional medication (e.g., diclofenac sodium, ibuprofen) with head-to-head comparison [23–25] or add-on design [26, 27]. Another randomized trial found that compared with fenbid solely, XFZY decoction plus fenbid had a better pain intensity relief [28]. But in the review [21], none of the included trials used a placebo as a control or with blinding of participants and personnel. Moreover, most of the previous trials appeared to be of low methodological quality. So far, XFZY’s efficacy lacks evidence from RCTs with rigorous methodology. Thus, we have designed a multicenter, participant- and personnel-blinded, randomized placebo-controlled trial to evaluate the efficacy and safety of XFZY for PD with *QBP*.

## Methods/design

### Aim

The aim is to evaluate the efficacy and safety of XFZY for PD patients with *QBP* in Chinese medicine (CM).

### Design and setting

**Fig. 1:**
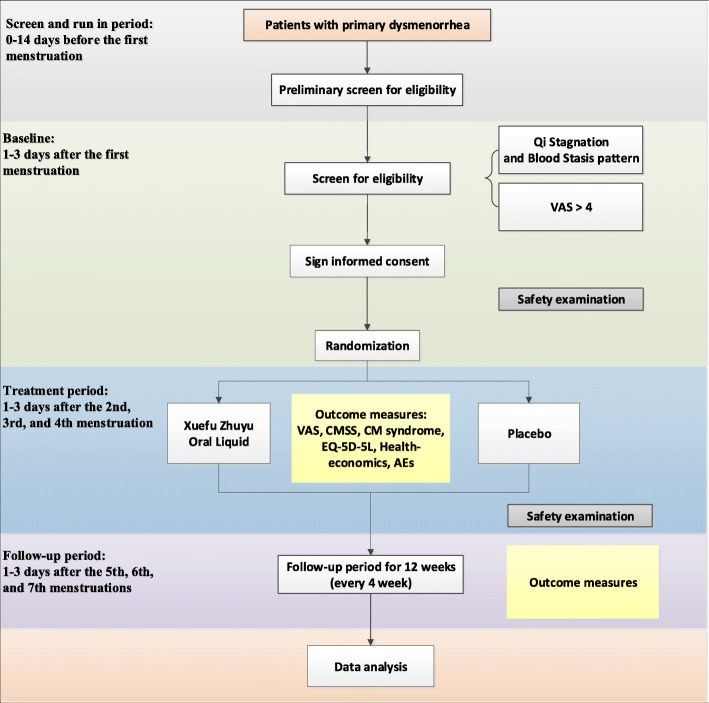
Flow diagram. VAS, visual analog scale; CMSS, Cox Menstrual Symptom Scale; AEs, adverse events

This is a multicenter, randomized, placebo-controlled trial. It will be conducted in 6 university hospitals in different Chinese provinces. A flow chart of this trial is provided in Fig. 1. The trial consists of a treatment period of 3 menstrual cycles and a follow-up period of 3 menstrual cycles. After providing written informed consent, eligible patients will be randomized to receive either XFZY or placebo at a ratio of 1:1.

The trial’s design and conduction will adhere to the Declaration of Helsinki and have been approved by the ethics committees at Guangdong Provincial Hospital of Chinese Medicine (No. BF2019-175-01) and each center. The trial was registered at the Chinese Clinical Trial Registry (No. ChiCTR1900026819) on 23 October 2019.

### Participants

#### Diagnostic criteria

##### PD diagnostic criteria

The diagnostic criteria for dysmenorrhea are based on primary dysmenorrhea consensus guidelines (2017) [5]. PD is characterized by crampy, suprapubic pain before or after the onset of menstrual bleeding. Its symptoms peak with maximum bleeding and may persist for 2–3 days. It is often accompanied by diarrhea, nausea and vomiting, fatigue, dizziness, headache, or even syncope and fever. In this RCT, abdominal ultrasound and gynecological examination will be conducted to exclude secondary dysmenorrhea and other causes of menstrual pain.

##### QBP diagnostic criteria

QBP diagnosis in CM is based on a scale which has been verified with validity and reliability [29]. Table 1 shows the symptoms and signs evaluated with the diagnostic scale.

**Table 1:** CM syndrome diagnostic scale for QBP [12]

Symptoms/signs	Yes	No	Score
Pain	9	0	
Irritability/depression	16	0	
Distending pain	2	0	
Scurry pain	6	0	
Chest distress	0.5	0	
Lumps in body	7	0	
Petechia in the tongue	4	0	
Purplish tongue	1	0	
Unsmooth pulse	4	0	
Deep pulse	2	0	
Total score If ≥ 20 points, it is diagnosed as QBP			

Pain includes stomachache, abdominal pain, low back pain, dysmenorrhea, breast pain, limb pain, etc.

*CM* Chinese medicine, *QBP* Qi stagnation and blood stasis pattern

##### Inclusion criteria

The following are the inclusion criteria:

1. Meet the diagnostic criteria for PD and QBP in CM
2. Aged 18 to 35 years
3. Menstrual cycle (21 to 35) days
4. Pain visual analog scale (VAS) score > 4
5. Signed informed consent

##### Exclusion criteria

The following are the exclusion criteria:

1. Secondary dysmenorrhea confirmed by gynecological ultrasound or caused by pelvic inflammation, endometriosis, cervix tumor, endometrial polyp, or other ailments
2. Severe primary cardiovascular, liver, kidney, or blood disease; mental illness (schizophrenia, epilepsy, alcoholism, anorexia, and/or a history of serious mental illness and those taking antidepressants, antiserotonin, barbiturates, or psychotropic drugs); or other severe primary diseases
3. Lactating and pregnant women or women who have recently been preparing for pregnancy
4. Allergy to herbal ingredients in the study
5. Participants in other clinical trials
6. Those who have been treated with hormone drugs over the last 3 months
7. Self-Rating Anxiety Scale (SAS) ≥ 60 or Self-Rating Depression Scale (SDS) > 62

### Recruitment

Recruitment advertisements will be posted on hospital notice boards and the WeChat (Shenzhen Tencent Computer System Co. Ltd., Guangdong, China) messaging client to recruit potential participants. Outpatient clinical staff will also screen and refer potential patients who will then access the study information. Interested PD patients can register for this trial by contacting researchers. Researchers will then arrange screening visits with potential participants 0–14 days before their onset of the next menstruation. Participants with PD will be enrolled at one of the following 6 hospitals in 4 different Chinese provinces: (1) Affiliated Hospital of Liaoning University of Traditional Chinese Medicine in Liaoning Province, (2) Guangdong Provincial Hospital of Chinese Medicine in Guangdong Province, (3) The Second Affiliated Hospital of Liaoning University of Traditional Chinese Medicine in Liaoning Province, (4) The First Affiliated Hospital of Heilongjiang University of Chinese Medicine in Heilongjiang Province, (5) The First Hospital of China Medical University in Liaoning Province, and (6) Affiliated Hospital of Inner Mongolia University for the Nationalities in Inner Mongolia Province. A signed consent form will be obtained from every participant who is eligible and willing to participate in the trial.

### Randomization and allocation concealment

A center-stratified and permuted block randomization sequence will be generated by SAS 9.2 (SAS Institute Inc., Cary, USA) and performed by the Institute of Basic Research in Clinical Medicine, China Academy of Chinese Medical Sciences (IBRCM). Eligible participants will be randomly allocated to either the XFZY group or the placebo group at a ratio of 1:1 through the Interactive Web Response System, developed by IBRCM. The randomization sequence will be strictly confidential and maintained by IBRCM. All participants, investigators, outcome assessors, statisticians, and other staff involved in this trial will have no chance to know the treatment allocation until the end of the study.

### Blinding

All participants, investigators, statisticians, pharmacists, and other staff will be blinded to the treatment assignments throughout the trial. The placebo will be identical to the XFZY in appearance, weight, taste, smell, and packaging. When serious adverse events (SAEs)/adverse reactions (SARs) occur or a subject’s condition deteriorates, and it is necessary to know their specific treatment so as to take emergency rescue, then blinding will be uncovered and participants and/or investigators will know the specific treatment. The reason, date, and results of the unblinding will be recorded in a case report form (CRF).

### Interventions

#### Experimental group

Participants in the experimental group will take XFZY orally three times per day (20 ml each dose) for 14 days before the onset of menstruation. The treatment period will last 3 menstrual cycles (XFZY will be administered for 14 days during each menstrual cycle). After treatment, there will be a 12-week follow-up.

The XFZY will be manufactured by Jilin Aodong Yanbian Pharmaceutical Co. Ltd. (Jilin Province, China) according to the requirements of the Good Manufacturing Practice (GMP). XFZY is composed of 11 herbs: peach kernel, safflower, *Rehmannia glutinosa*, ligusticum chuanxiong, red peony root, radix platycodonis, radix platycodonis, radix bupleurum, bran fried fructus aurantii, licorice, and *Angelica sinensis*.

#### Control group

Patients in the control group will receive a placebo with no active ingredients, but consistent with XFZY in terms of dosage, appearance, smell, taste, and texture. It will also meet the hygiene inspection requirements. The placebo’s primary ingredients are honey, white granulated sugar, fried brown sugar, bitterants, ginseng essence, natural edible pigments, and food antiseptic. The placebo will also be taken orally (20 ml each dose) three times per day for 14 days before the onset of menstruation for 3 menstrual cycles. The placebo is also manufactured by Jilin Aodong Yanbian Pharmaceutical Co. Ltd. in line with the GMP requirements.

Both XFZY and the placebo will be labeled and packaged based on a randomization sequence by Jilin Aodong Yanbian Pharmaceutical Co. Ltd. under the supervision of IBRCM.

A box containing a 14-day dose of either XFZY or placebo will be distributed to subjects in the pharmacy department or clinic office every menstrual cycle, from visit 1 (menstrual cycle 1) to visit 4 (menstrual cycle 4).

#### Discontinuing interventions

Participants will be free to withdraw from this clinical study at any time and for any reason. The reasons and time for discontinuing will be recorded on CRFs, reported, and analyzed. Subjects will be terminated from the trial if the following conditions occur: (1) PD aggravates and progresses aggressively during the treatment period and investigators decide the patient needs to withdraw; (2) during the research period severe systemic disease such as an active malignant tumor, decompensated cirrhosis, or hematopoietic systems diseases are found; (3) surgery is needed due to acute abdomen, trauma, or other diseases during the treatment period; (4) voluntary withdrawal from the trial; or (5) serious complications occur, and treatment is needed.

#### Compliance assessment

Patients will be asked to record a diary of intervention medicine taken, and to return any remaining medicine. The compliance rate will be calculated with the following formula: Compliance rate = (actual dosage /expected dosage) × 100%. Patients with compliance rates equal to or exceeding 80% will be considered to have high compliance.

#### Rescue and concomitant treatment

When patients undergo unbearable pain during menstruation, painkillers (Fendid) will be given according to doctors’ orders, and the concomitant medication will be recorded. Other drugs that alleviate menstrual pain will be prohibited, such as oral contraceptives. Chinese herbal medicines that have the effect of moving *Qi* and activating the *blood* will also be prohibited.

### Outcome measures

Outcome measurement time points are provided in Table 2.

**Table 2:**
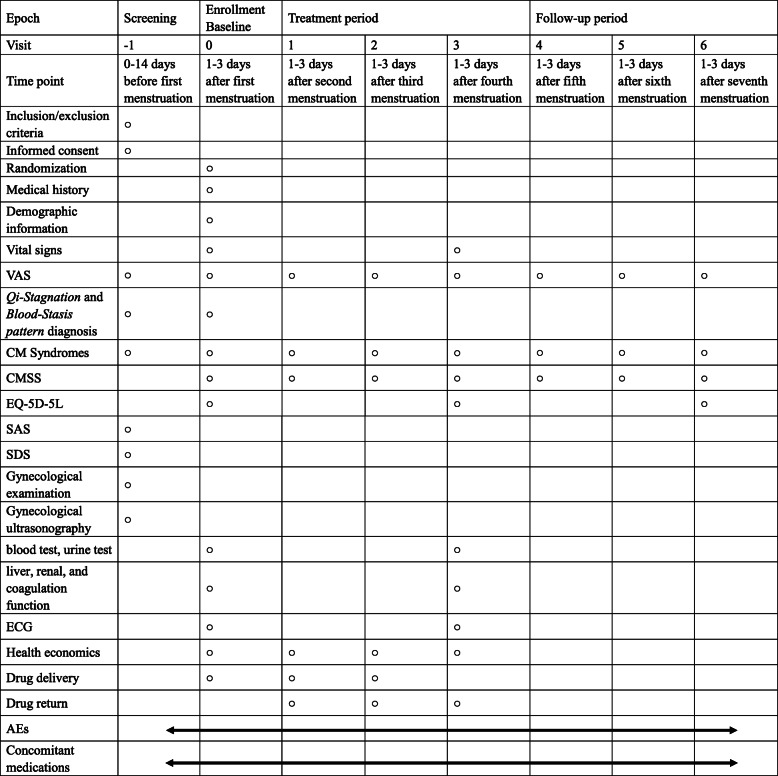
Schedule for enrollment, interventions, and assessments

*VAS* visual analog scale, *CMSS* Cox Menstrual Symptom Scale, *SAS* Self-Rating Anxiety Scale, *SDS* Self-Rating Depression Scale, *ECG* electrocardiograph, *AEs* adverse events

#### Primary outcome

The primary outcome is change in the mean pain intensity as measured by the visual analog scale (VAS), from baseline to the end of treatment. The VAS rates the severity of menstrual pain from 0 to 10 cm (no pain to the most serious pain imaginable) [30]. Participants will self-rate their mean pain intensity using VAS at baseline and each visit.

#### Secondary outcomes

The secondary outcomes are as follows:

- The change in menstrual pain duration
- The change in peak pain intensity as measured by VAS
- CM syndrome change
- Cox Menstrual Symptom Scale (CMSS) [31]
- Quality of life, as measured by EQ-5D-5L [32]
- Cumulative painkiller consumption
- Health economics

Participants will self-rate their peak pain intensity using VAS, report menstrual pain duration, and fill out CMSS at baseline and each visit. Investigators will evaluate the subjects’ CM syndrome at baseline and each visit. EQ-5D-5L will be completed by the participant at baseline, the end of treatment, and the end of the follow-up period. Health economics will be assessed at baseline and each visit of treatment duration. Investigators will look through diaries filled out by participants to confirm the painkiller consumption at every visit (see Table 2).

### Safety assessment

Adverse events (AEs) experienced by the patient at any point in the trial will be reported to the investigators and recorded on the CRFs. In addition to participant self-report, physicians will inquiry the participants to seek and record any AEs that happened in the duration of the trial. Besides, abnormal laboratory findings with clinical significance will also be recorded as AEs. AE details include the time of occurrence, severity, causality to the intervention, whether the event was serious, action taken, and AE outcome. The WHO Uppsala Monitoring Centre System for Standardized Case Causality Assessment will be used to assess the causality between AEs and intervention [33]. XFZY may cause AEs such as upset stomach [34], dizziness, rash, insomnia, fever, or toothache [35]. SAEs must be reported to both the Data and Safety Monitoring Committee (DSMC) and the Ethics Committee within 24 h.

### Sample size calculation

At present, no trial has compared XFZY for PD to a placebo. Therefore, we referred to Chou’s trial which studied the effect of XFZY decoction on PD and used VAS score as an outcome measure. According to Chou’s study, the mean change from baseline to the end of treatment was 4.9 ± 0.94 (mean ± SD) [34]. XFZY is a modified dosage of Xuefu Zhuyu Decoction, and the treatment in Chou’s study was added or subtracted on the basis of the pattern. Thus, we assumed that the mean change in VAS should be 3.0 ± 0.94 in the XFZY group. In a previous trial, the mean change for VAS in the placebo group was 0.88 ± 1.64 [36]. The minimal clinically important difference (MCID) for VAS was 1.6 on acute abdominal pain [37]. In this trial, the mean changes in VAS score after 12 weeks of treatment (3 menstrual cycles) for both the XFZY group and the placebo group were assumed to be 3 ± 0.94 and 0.88 ± 1.64, respectively. We set *α* to 0.025 and *β* to 0.2 and calculated as if the sample size was 105 in each group. Calculations were done with PASS (version 11.0, NCSS, LLC. Kaysville, UT, USA). Anticipating a dropout rate of 15%, we will require a sample size of 124 for each group.

### Data management and quality control

Before recruitment, investigators, assessors, and research assistants will attend a training workshop to ensure they adhere to the study protocol and are familiar with the administration process. Also, standard operating procedures (SOPs) will be drafted and sent to the entire research team.

CRFs, PROs, and other resource trial data will be checked by the investigators themselves and by clinical research associates (CRA) from an independent department at Jilin Aodong Yanbian Pharmaceutical Co. Ltd. They will monitor all collected data. The GPHCM Department of Scientific Research and the Office of National Key Technology R&D Program for the Thirteen Five-year Plan of the Ministry of Science and Technology, China, will inspect the trial. The DSMC will be established before the trial in order to monitor the safety events throughout.

Data will be collected according to SOPs and entered into an electronic data capture (EDC) system developed by IBRCM. Traces of any data modification will be required to be retained. A data manager will check all the data without knowing the treatment allocation and will notify the investigator of any discrepancies. The database will be locked after all data have been checked and cleaned.

### Statistical analysis

Data will be processed with PASW Statistics 18.0 (IBM SPSS Inc., Armonk, New York, USA) and SAS (SAS Institute Inc., Cary, USA). Statistical analysis will be performed by qualified statisticians who are independent from the research team and are blinded to treatment allocation. Two-tailed *P* values < 0.05 will be considered statistically significant. The analysis will be based on the intent-to-treat (ITT) and the per-protocol (PP) principle. The ITT population will include all participants randomized to this trial. The PP population will only include participants with neither major protocol deviations nor low compliance. Missing data will be replaced in accordance with the principle of multiple imputations. There is no plan for any interim analysis. Efficacy assessment will be analyzed by ITT and PP population, simultaneously. If these two results are inconsistent, the ITT analysis will be considered the primary result.

Demographic and other baseline data will be presented by descriptive statistics. An independent *t* test will compare the change in the mean pain intensity as measured by VAS from baseline to the end of the treatment, between the two groups’ primary outcomes. Superiority will be confirmed by 95% confidence intervals. For secondary outcomes, continuous and categorical data will be compared between the two groups with either a *t* test, chi-square test or Fisher’s exact test. Frequency, mean, standard deviation (SD), median, and range of secondary outcomes will be summarized. To analyze the factors affecting the outcome change at each time point, either a mixed-effects or a linear or logistic regression model will be performed adjusting for baseline characteristics such as age, menstrual pain severity, cumulative painkiller consumption, and other variables. The repeated measure analysis of variance will be conducted to distinguish the treatment effect and time effects. Subgroup analysis will be performed based on disease severity and age.

Safety will be assessed by AEs and ADRs and will be presented with descriptive statistics for each group. Frequency differences for AEs and ADRs will be compared with a chi-square test or Fisher’s exact test.

## Discussion

This protocol describes a randomized, placebo-controlled, double-blind, parallel-group multicenter trial to investigate the efficacy and safety of XFZY for primary dysmenorrhea patients with *QBP* according to CM. Validated evaluation tools will be used to assess dysmenorrhea severity. To our knowledge, this is the first clinical study to investigate the efficacy of XFZY for PD compared to a placebo. The use of a placebo has the advantage of preventing expectation biases resulting from inadequate blinding. Blinding of participants, personnel, and outcome assessors may reduce the risk that knowledge of which intervention was received, rather than the intervention itself, affects outcomes. When blinding is effective, a similar amount of attention and ancillary treatment between groups can be ensured. It is especially important to apply blinding to the outcome assessors to assess subjective outcomes, such as the degree of postoperative pain [38].

This study may be limited in that 3 months of treatment duration may be insufficient for complete PD pain relief. However, a systematic review of XFZY for PD [21] found that the treatment duration was 3 months (menstrual cycles) for all of the included trials, with only one exception [39]. Therefore, we believe that 3 months is a sufficient duration for observing the preliminary clinical effects of XFZY on PD and that an RCT is needed to evaluate the long-term effects.

In sum, this trial is the first study designed to demonstrate the efficacy and safety of XFZY in treating PD patients, compared with a placebo.

### Trial status

The protocol version is V1.0/20190920. The trial started in January 2020 and is currently recruiting patients. It is expected to be complete in March 2021.

## Data Availability

Data sharing is not applicable to this article as no datasets were generated or analyzed during the current study. However, the data will be available from the corresponding authors on reasonable request after study completion.

## Abbreviations

AEs: Adverse events
ADRs: Adverse drug reactions
CFDA: China Food and Drug Administration
CM: Chinese medicine
CMSS: Cox Menstrual Symptom Scale
CRF: Case report form
CRA: Clinical research associates
EDC: Electronic data capture
DSMC: Data and Safety Monitoring Committee
GPHCM: Guangdong Provincial Hospital of Chinese Medicine
GMP: Good Manufacturing Practice
IBRCM: China Academy of Chinese Medical Sciences
ITT: Intent-to-treat principle
NSAIDs: Non-steroidal anti-inflammatory drug
PD: Primary dysmenorrhea
PP: Per-protocol principle
QBP: Qi stagnation and blood stasis pattern
SD: Standard deviation
SAEs: Serious adverse events
SARs: Serious adverse reactions
SOPs: Standard operating procedures
VAS: Visual analog scale
XFZY: Xuefu Zhuyu
